# A Case of Epidermal Cyst in the Retrorectum Safely Resected by a Combined Laparoscopic Approach

**DOI:** 10.1111/ases.70068

**Published:** 2025-04-23

**Authors:** Yusuke Tanaka, Hiroaki Kasashima, Tatsunari Fukuoka, Ken Yonemitsu, Yuki Seki, Kenji Kuroda, Yuichiro Miki, Mami Yoshii, Tatsuro Tamura, Masatsune Shibutani, Takahiro Toyokawa, Shigeru Ree, Kiyoshi Maeda

**Affiliations:** ^1^ Department of Gastroenterological Surgery Osaka Metropolitan University Graduate School of Medicine Osaka Japan; ^2^ Department of Gastroenterological Surgery Tsukazaki Hospital Himeji Japan

**Keywords:** epidermal cyst, laparoscopic, presacral, retrorectum

## Abstract

The surgical indications and optimal approach for retrorectal tumors remain unclear due to their rarity and the anatomical complexity of the presacral space. We report the case of a 48‐year‐old man in whom a retrorectal mass was incidentally detected on abdominal and pelvic computed tomography. Magnetic resonance imaging demonstrated a cystic lesion measuring 32 × 12 × 20 mm, with low signal intensity on T1‐weighted and high signal intensity on T2‐weighted images. Given the difficulty of establishing a definitive diagnosis and the potential risk of infection or tumor seeding with biopsy, primary surgical resection was selected. A combined laparoscopic transabdominal and trans‐sacral approach enabled precise dissection under enhanced visualization of the pelvic anatomy, ensuring safe and complete tumor excision. The patient's postoperative course was uneventful, and he remained recurrence‐free at the 1‐year follow‐up. This case highlights the pivotal role of laparoscopy in facilitating the safe resection of retrorectal tumors.

**Abbreviations**
CT: computed tomography
MRI: magnetic resonance imaging

## 1 Introduction

There are currently no established criteria for the surgical resection of retrorectal tumors (RRTs). In this report, we present a case in which a tumor was successfully resected using a combined trans‐sacral and laparoscopic approach.

## 2 Case Presentation

A 48‐year‐old male with no subjective symptoms was referred to our hospital following the incidental detection of a tumor on the anterior sacrum and retrorectum during an abdominal and pelvic CT scan. The cyst was neither visible nor palpable on physical examination. Blood tests revealed no abnormalities in blood counts, coagulation parameters, or tumor markers. Lower gastrointestinal endoscopy identified several small polyps in the ascending, transverse, and sigmoid colon; however, no malignant findings or evidence of extramural rectal compression were observed.

**FIGURE 1:**
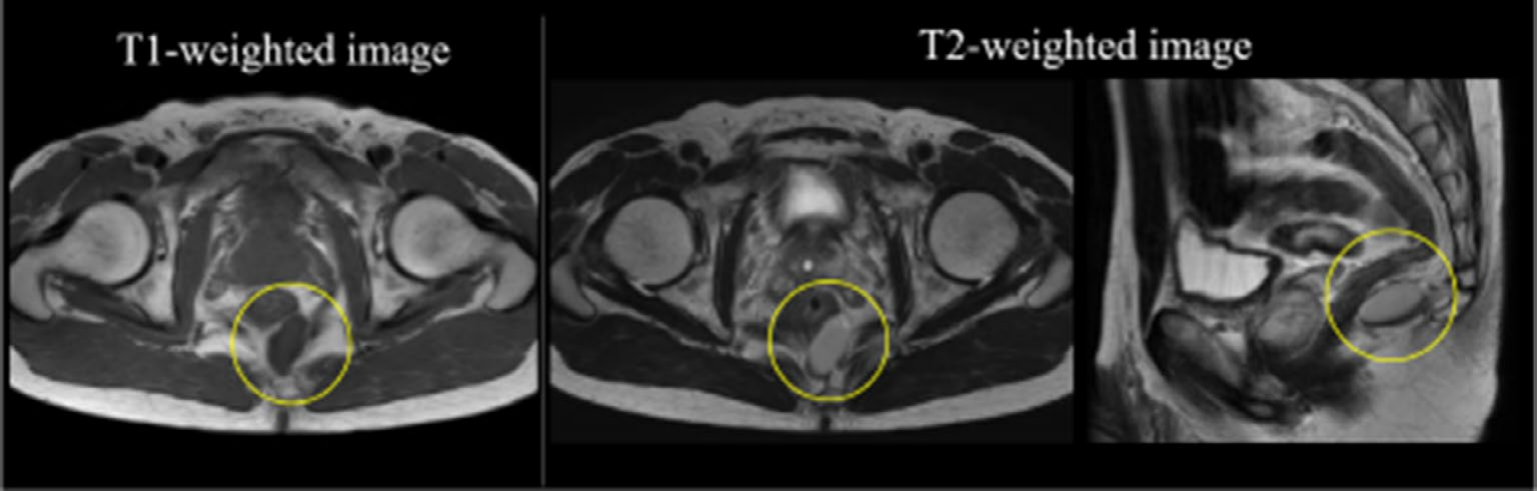
Abdominal MRI shows a cystic lesion measuring 32 × 12 × 20 mm, characterized by low signal intensity on T1‐weighted images and high signal intensity on T2‐weighted images.

An MRI scan of the abdominopelvic region revealed a cystic lesion measuring 32 × 12 × 20 mm, characterized by low signal intensity on T1‐weighted images and high signal intensity on T2‐weighted images (Figure 1). Due to the difficulty in accurately characterizing the lesion based on imaging alone, we opted against performing puncture cytology or biopsy, given the potential risk of infection or tumor seeding in the case of malignancy. Instead, we proceeded with surgical resection. The tumor was successfully removed using a combined laparoscopic and trans‐sacral approach.

**FIGURE 2:**
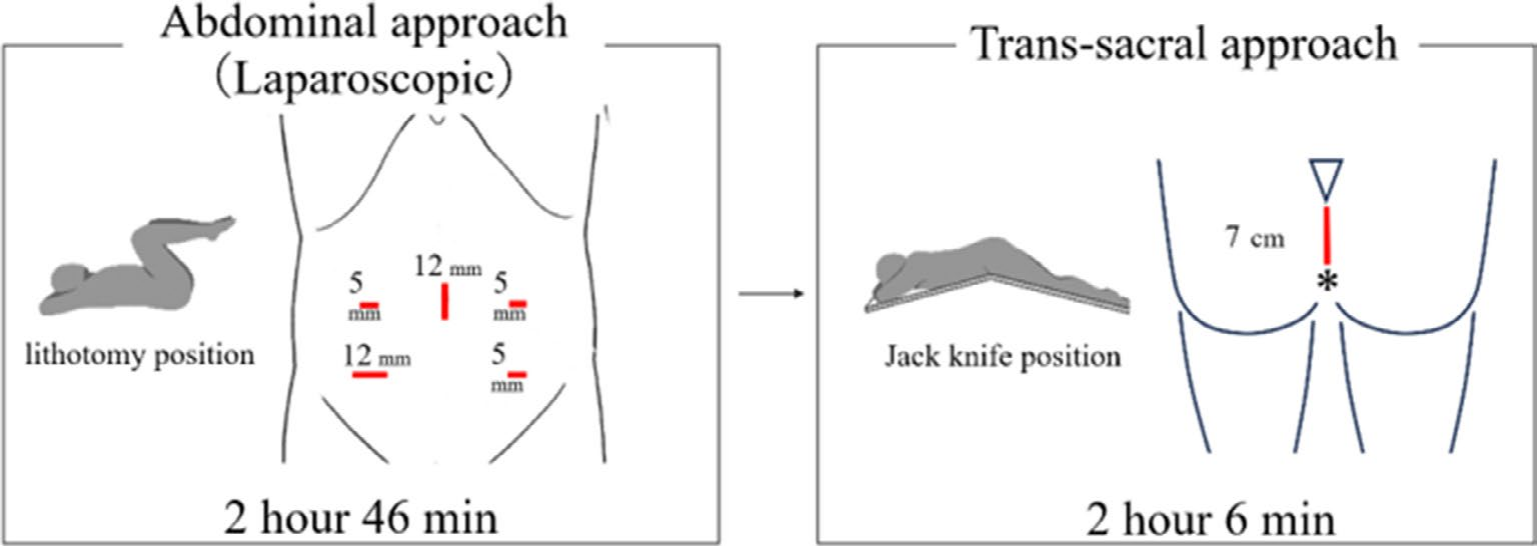
The abdominal approach was performed first, followed by the trans‐sacral approach.

Laparoscopic manipulation was performed with the patient in the lithotomy position. A 12 mm camera port was inserted through the umbilicus, and a total of five ports were utilized (Figure 2). The mesorectum was dissected to expose the posterior rectal space and the posterior wall of the rectum, followed by lateral dissection until the levator ani muscle was identified. However, the tumor was not visible within the mesorectum and could not be identified on the anterior surface of the sacrum. Even with the use of intracorporeal ultrasound, confirming the lesion proved challenging. Consequently, laparoscopic manipulation was discontinued, and half‐gauze was placed at the deepest point of dissection to facilitate subsequent trans‐sacral resection.

**FIGURE 3:**
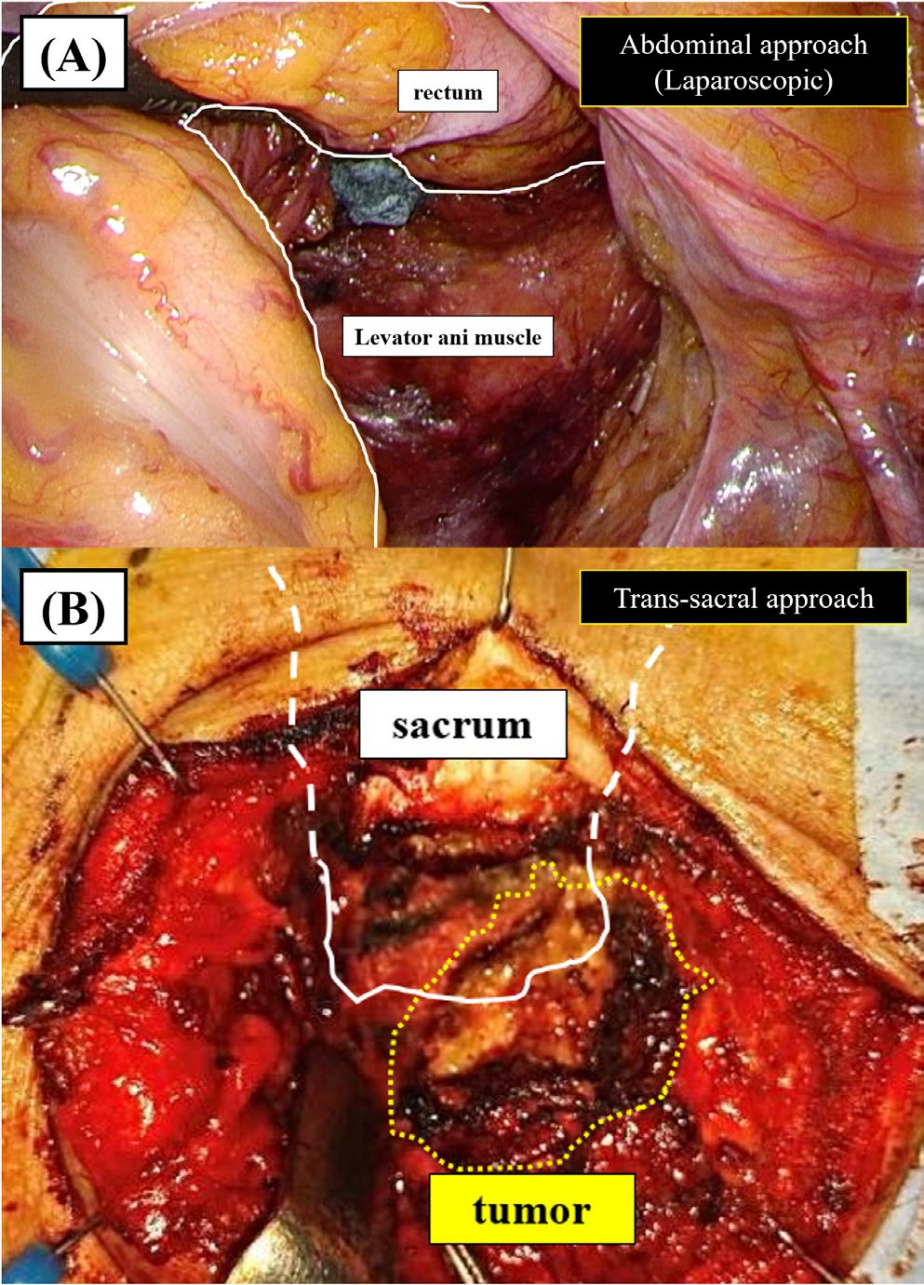
(A) The abdominal approach was performed until the levator ani muscle was identified; however, the tumor was not visualized. Gauze was placed at the deepest point of dissection. (B) During the trans‐sacral approach, the gauze placed during the abdominal procedure was identified. The tumor was successfully removed without causing rectal injury.

The patient was repositioned to the Jackknife position, and the lesion's location was confirmed using body surface ultrasound. A 7 cm midline incision was made cephalocaudally in the perineal region, and careful dissection was performed around the tumor under ultrasound guidance. During the dissection, the tumor was successfully excised without damaging the rectum by passing it through the abdominal cavity, using the previously placed half‐gauze as a reference. Additionally, due to tumor adhesion to the coccyx, partial resection of the coccyx was performed (Figures 3 and S1).

The excised specimen contained a white, muddy fluid. Histopathological examination revealed a cystic lesion lined with squamous epithelium, without skin adnexa in the cyst wall, leading to a diagnosis of an epidermal cyst. The patient had an uneventful postoperative course and was discharged on the seventh postoperative day. At the 1‐year follow‐up, no signs of recurrence were observed.

## 3 Discussion

The space between the rectum and the sacrum undergoes complex developmental changes during fetal growth, involving all three germ layers, which can lead to the formation of various masses [1]. RRTs are rare [2]; these tumors are often asymptomatic until they reach a significant size and are frequently discovered incidentally. RRTs are classified into congenital, inflammatory, neurogenic, osteogenic, and other types, with congenital tumors being the most common, accounting for approximately 39% of cases [3]. Hawkins et al. categorized congenital retrorectal masses as developmental cysts and further classified them histologically into three subtypes: dermoid cysts, epidermal cysts, and mucus‐secreting cysts. Both dermoid and epidermal cysts are lined by squamous epithelium and contain a white atheromatous material, whereas mucus‐secreting cysts are remnants of the embryonic tail gut and are lined by columnar epithelium [4]. Preoperative differentiation of RRTs remains challenging due to the lack of distinct imaging characteristics. Additionally, biopsy is generally avoided due to the risk of tumor seeding, abscess formation, and inflammation [5]. Prior to surgery, several differential diagnoses were considered based on imaging findings. These included posterior rectal cysts such as epidermoid cysts, epidermoid cysts, and mucous cysts, as well as other possible masses such as abscesses and benign tumors. Given that there are reports of squamous cell carcinoma arising from epidermal cysts, including a case by Kim et al. [6], early surgical resection is recommended for both diagnostic and therapeutic purposes.

Approaches to the resection of RRTs include trans‐sacral, transabdominal (including laparoscopic), or a combination of both. The choice of surgical technique depends on the tumor's location and size. Generally, the transabdominal approach is recommended for tumors located above the S2–S3 level, while the trans‐sacral approach is preferred for tumors below this level. Some reports suggest that the trans‐sacral approach is most effective for tumors located below S3, particularly for lesions measuring less than 10 cm, situated below S4, and without invasion of surrounding structures [7]. Laparoscopic surgery offers several advantages, including smaller incisions, reduced postoperative pain, and an enhanced field of view, which improves visualization of anatomical structures. Due to its familiarity and effectiveness, this approach is increasingly utilized in colorectal surgery. Aubert et al. [8] conducted a multicenter study of 270 patients who underwent RRT resection in France between 2000 and 2019. Their analysis found no significant differences in morbidity, severity of surgical complications, reintervention rates, or readmission rates between the laparoscopic approach and the modified Kraske procedure. However, postoperative ileus was more common with the laparoscopic approach, while the modified Kraske procedure had a higher incidence of wound abscesses. Galán Martínez et al. suggested that laparoscopic surgery should be considered even for deeper tumors, as it is associated with shorter hospital stays and fewer postoperative complications compared to the trans‐sacral approach [9]. In recent years, laparoscopic surgery has advanced significantly, and robotic‐assisted procedures have also become increasingly widespread. The pursuit of strategies to sufficiently reduce the risk of rectal injury has preceded and contributed to the development of laparoscopic techniques.

In our case, although the tumor measured 32 mm in diameter and was located deeper than the S3 level, we initially utilized a laparoscopic approach. While laparoscopic resection was not feasible, the tumor was safely removed using a trans‐sacral approach, guided by the half‐gauze placed during the laparoscopic procedure. The placement of the half‐gauze in this procedure may play an important role in improving surgical outcomes. First, in terms of rectal protection: During dissection, particularly within the confined space of the pelvis, inadvertent injury or irritation of surrounding tissues can often occur. The half‐gauze serves as a protective barrier, preventing direct contact between surgical instruments and delicate structures, thereby reducing the risk of unintended injury. In particular, in cases of rectal injury, repair via the trans‐sacral approach is challenging; thus, the use of half‐gauze was effective in preventing rectal damage. Second, in preventing tumor cell seeding: The risk of tumor cell dissemination is always a concern during tumor resection. This is especially true in narrow or delicate areas such as the sacral region. The gauze helps minimize the spread of malignant cells by absorbing and containing any potential spillage of tumor contents.

Yang et al. [10] reported malignant transformation in 1 out of approximately 60 cases of presacral epidermal cysts. While the exact mechanism remains unclear, chronic inflammation of the cyst is thought to stimulate the cyst epithelium, potentially leading to malignant transformation. Kim et al. reported cases of squamous cell carcinoma arising from epidermal cysts. They followed 3290 patients who underwent surgical resection for suspected epidermal cysts between 2005 and 2023 and identified nine patients who were pathologically diagnosed with squamous cell carcinoma originating from epidermal cysts. A retrospective analysis of these nine cases revealed a mean latency period of 15.4 years [6]. Currently, 1 year has passed since the surgery, with no signs of recurrence. This case demonstrates that a combined laparoscopic and trans‐sacral approach can facilitate safe and effective tumor resection in the retrorectal space while avoiding rectal injury.

We report a case in which a RRT was safely resected using a combined laparoscopic and trans‐sacral approach, successfully avoiding rectal injury.

## Author Contributions

Y.T. and H.K. drafted the manuscript. T.F., K.K., Y.M., M.Y., T.T., M.S., S.R., and T.T. participated in its design and coordination and helped to draft the manuscript. K.M. contributed to the critical revision. All authors read and approved the final manuscript. All authors agree to be responsible for all aspects of the study.

## Ethics Statement

The study procedures adhered to the tenets of the Declaration of Helsinki.

## Consent

Written informed consent was obtained from the patient for publication of this case report and any accompanying images.

## Conflicts of Interest

The authors declare no conflicts of interest.

## Supporting information


**Figure S1.** The position in relation to the indwelling half‐gauze, rectum, and sacrum.

## Data Availability

Data sharing is not applicable to this article as no datasets were generated or analyzed during the current study.
