# Supplementary figures and images for: A Case of Epidermal Cyst in the Retrorectum Safely Resected by a Combined Laparoscopic Approach
Source: Asian J Endosc Surg. 2025 Apr 23;18(1):e70068. doi: 10.1111/ases.70068 (PMC12018787; doi:10.1111/ases.70068)

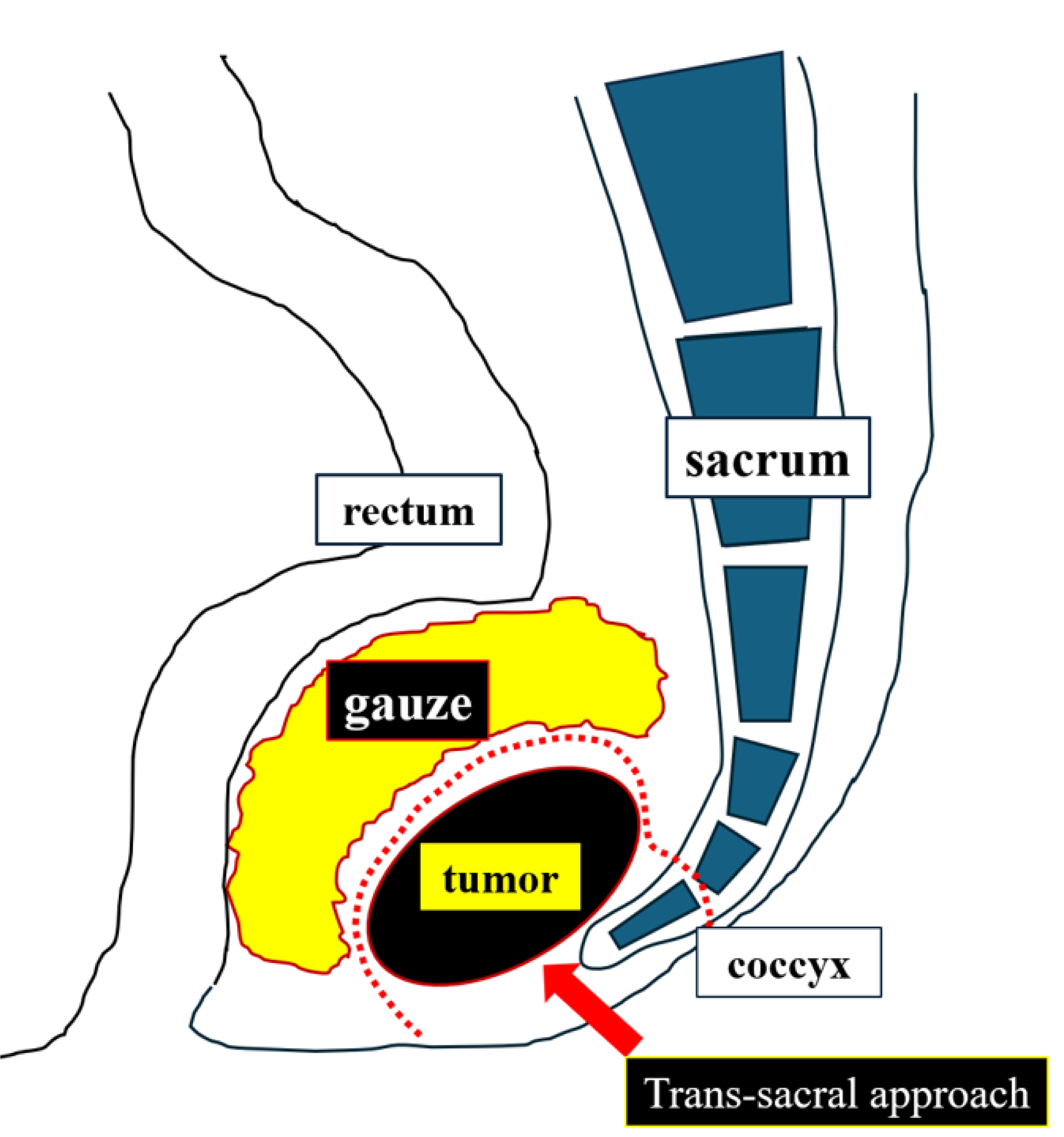

Supplement: Supplementary file 1 — Figure S1. The position in relation to the indwelling half‐gauze, rectum, and sacrum. [file ASES-18-e70068-s001.tif]
